# The thirty-second sit-to-stand test as a predictor of postoperative complications of lung resection: a real-world study

**DOI:** 10.36416/1806-3756/e20240342

**Published:** 2025-03-18

**Authors:** Inês Fernandes Pedro, Diana Organista, Teresa Seguro, Mariana Maia Silva, Fátima Rodrigues

**Affiliations:** 1. Serviço de Pneumologia, Unidade Local de Saúde Santa Maria, Lisboa, Portugal.; 2. Fundação Champalimaud, Lisboa, Portugal.; 3. Unidade Local de Saúde de Amadora/Sintra, Amadora, Portugal.; 4. Faculdade de Medicina, Universidade de Lisboa; Instituto de Saúde Ambiental

## TO THE EDITOR:

Although the prevalence of lung cancer is on the rise, scientific and technological advances have improved diagnosis and treatment, with thoracic surgery playing a growing role in the latter. Because postoperative complications increase patient morbidity and mortality, as well as the duration and cost of hospitalization, careful preoperative evaluation must be performed in order to identify patients at greater risk of postoperative complications. Although advances in surgical techniques have made it possible to reduce postoperative complications, they still occur in 6.8-30% of patients undergoing noncardiac thoracic surgery.^1^ Therefore, additional tools are needed to predict the risk of postoperative complications.^2^

Postoperative complications have long been associated with exercise capacity, specifically peak oxygen consumption, the risk of complications and mortality being lower in patients with higher peak oxygen consumption.^3^ Strength and speed are components of exercise capacity and also appear to be associated with postoperative complications. The thirty-second sit-to-stand test (30STS) is a simple field-based exercise test that assesses strength and speed and might be useful in assessing the risk of postoperative complications in candidates for lung resection. 

We conducted a prospective cohort study of adult patients admitted to the Department of Thoracic Surgery of *Hospital Pulido Valente*, located in the city of Lisbon, Portugal. The patients included in the study underwent lung resection between May 1, 2021 and September 7, 2022, and we assessed predictors of postoperative complications.

We collected data on the following: age; sex; BMI; smoking history; comorbidities; level of physical activity; pulmonary function tests; condition requiring surgery (including benign and malignant neoplasms, as well as infections); surgical approach; type of lung resection; length of hospital stay (in days); duration of chest tube drainage (in days); postoperative complications; peak cough flow; and 30STS results. The 30STS was fully standardized^4^ and was supervised by a physiotherapist, being performed preoperatively and within 24 h of chest tube removal. Data on complications were obtained from patient medical records and validated by the study investigator, who was present during the entire study period. The sample size was estimated at 100 patients on the basis of the rate of complications reported elsewhere,^5^ with a 95% confidence interval and a 5% margin of error, with the use of the Raosoft^®^ sample size calculator (Raosoft, Inc., Seattle, WA, USA). The study was approved by the local research ethics committee (Protocol no.118/21). 

The study sample was divided into two groups of patients: those with and those without postoperative complications. All statistical analyses were performed with the IBM SPSS Statistics software package, version 25.0 for macOS (IBM Corporation, Armonk, NY, USA), and a two-sided p-value of 0.05 was considered statistically significant for all tests. Categorical data were presented as frequencies and percentages, and continuous variables were presented as mean and standard deviation, as appropriate. Comparisons between continuous variables were analyzed with logistic regression, and comparisons between categorical variables were performed by the chi-square test. Optimal 30STS thresholds were determined by ROC analysis. A multivariate Cox regression model was created to include variables showing univariate association with complications. Missing data were subject to listwise deletion. 

A total of 101 patients were included in the present study. The sociodemographic and clinical characteristics of the study sample are summarized in Table 1. The postoperative complication rate was 22%, with 29 complications documented in 22 patients. The most common postoperative complications were pulmonary complications (n = 25; 86.2%). These included persistent air leak (n = 11), respiratory infection (n = 6), bronchopleural fistula (n = 5), atelectasis (n = 1), chylothorax (n = 1), and pneumothorax after chest tube removal (n = 1). Death was recorded in 2 patients (6.9%). Cardiovascular complications followed (n = 1; 3.4%), with 1 case of arrhythmia, as did technical complications (n = 1; 3.4%), with the need to convert to thoracotomy during thoracoscopic lobectomy (n =1). Most of the complications occurred in men (n = 22; 86.2%). 

**Table 1:** Sociodemographic and clinical characteristics of patients with and without postoperative complications of lung resection.^a^

	Total sample	Without postoperative complications	With postoperative complications	p
Sex, male; female	58 (57); 43 (43)	41 (52); 38 (48)	17 (77); 5 (23)	0.033
Age, years	66.1 ± 10 [37-86]	66.4 ± 9,8 [37-86]	66. 1 ± 9,9 [39-81]	0.262
BMI, kg/m^2^	25.8 ± 4.2 [16.3-37.5]	26.2 ± 4.4 [16.3-37.5]	24.5 ± 4.1 [18.1-30.9]	0.355
Smoking status, NS; FS; CS	34 (34); 20 (20); 47 (47)	29 (37); 36 (46); 14 (18)	5 (23); 11 (50); 6 (27)	0.220
Smoking history, pack-years	32.1 ± 37 [0-150]	27.8 ± 35 [0-120]	46 ± 33 [0-150]	0.055
Physical activity, IA; SA	80 (79); 21 (21)	62 (78); 17 (22)	18 (82); 4 (18)	0.733
FEV_1_, L	2.4 ± 0.7 [1-4]	2.3 ± 1 [1-3.99]	2.5 ± 0.7 [1.62-4]	0.441
DL_CO_, %	80.1 ± 19.2 [38.6-131]	83.4 ± 19.5 [38.6-131]	65.4 ± 17.8 [54-97]	0.005
DL_CO_/V_A_, %	84.4 ± 19.6 [46-136]	89.3 ± 19.6 [46-136]	64.8 ± 18.5 [48-90]	0.0002
Indication for surgery, benign neoplasm; infection; malign neoplasm	4 (4); 5 (5); 92 (91)	4 (5); 2 (2); 74 (97)	0 (0); 3 (14); 19 (86)	0.405
Surgical approach, thoracotomy; VATS	42 (42); 59 (58)	27 (34); 52 (66)	15 (68); 7 (32)	0.004
Type of lung resection				0.081
Enucleation	1 (1)	1 (1)	0 (0)	
Enucleation + atypical lung resection	1 (1)	1 (1)	0 (0)	
Pneumonectomy	2 (2)	2 (3)	0 (0)	
Bilobectomy	4 (4)	3 (4)	1 (5)	
Atypical lung resection	28 (28)	26 (33)	2 (9)	
Lobectomy	65 (64)	46 (58)	19 (86)	
Operative time, min	146.7 ± 70.8 [27-368]	136.5 ± 67.6 [27-327]	183.1 ± 59.9 [82-368]	0.010
Preoperative 30STS, total sample	11.6 ± 3.2 [2-22]	11.9 ± 3.1 [2-22]	10.5 ± 2.9 [5-18]	0.059
Preoperative 30STS, men	12.2 ± 2.9 [5-22]	13.0 ± 2.8 [8-22]	10.4 ± 2.6 [5-17]	0.008
Preoperative 30STS, women	10.8 ± 3.2 [2-18]	10.8 ± 3.2 [2-17]	10.6 ± 3.0 [6-18]	0.871

NS: nonsmoker; FS: former smoker; CS: current smoker; IA: insufficiently active; SA: sufficiently active; V_A_: alveolar volume; VATS: video-assisted thoracoscopic surgery; and 30STS: thirty-second sit-to-stand test. ^a^Data expressed as n (%) or mean ± SD [min-max].

In the study population as a whole, the mean length of hospital stay was 8.2 [3-28] days and the mean duration of chest tube drainage was 5.13 [1-25] days. In the subgroup of patients with postoperative complications, the mean length of hospital stay was 15 days and the mean duration of chest tube drainage was 11.6 days. The two patients who died during hospitalization were 65-year-old and 68-year-old men with COPD who had undergone lobectomy by thoracotomy for infectious and neoplastic diseases. 

After univariate analysis, sex, DL_CO_, DL_CO_/alveolar volume (V_A_), surgical approach, and operative time correlated with postoperative complications (Table 1). However, preoperative peak cough flow did not. 

**Figure 1:**
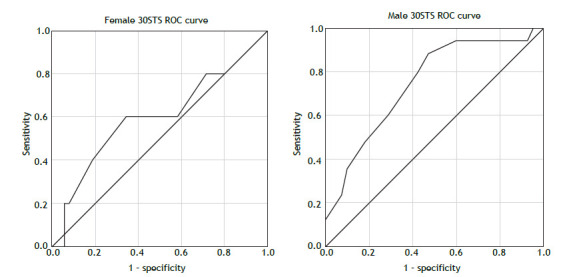
ROC analysis of the thirty-second sit-to-stand test (30STS) for female patients and male patients.

In the study population as a whole, 30STS showed a trend toward predicting complications (p = 0.059), being a good predictor of complications in the male population (p = 0.008). This might be due to the fact that there were significantly more men than women in the subgroup of patients with postoperative complications (Table 1). As can be seen in Figure 1, the optimal preoperative 30STS thresholds were 9.5 full stands for women (sensitivity, 60%; specificity, 66%) and 12.5 full stands for men (sensitivity, 88%; specificity, 53%). Thus, a preoperative test below the aforementioned thresholds increased the risk of complications by 43% (OR: 1.438; 95% CI, 1.150-1.797; p = 0.001). We obtained comparable results when analyzing 30STS as a percentage of the predicted values^6^: the test predicted postoperative complications in the study population as a whole (p = 0.029) and in the male population (p = 0.007). Performances below the thresholds of 52.5% for women (sensitivity, 60%; specificity, 83%) and 74.4% for men (sensitivity, 73%; specificity, 69%) increased the risk of complications by 60% (OR: 1.608; 95% CI, 1.173-2.206; p = 0.0001). 

In the multivariate analysis, DL_CO_/V_A_ and sex-specific 30STS results were the only variables that remained prognostic for postoperative complications (p = 0.011 and p = 0.015, respectively). In a subgroup of surgical patients selected on the basis of lung function, as assessed by current standards,^2^ DL_CO_/V_A_ retained its role in predicting postoperative complications. 

As far as we know, this is the first study to demonstrate an association between a field-based exercise test such as the 30STS and postoperative complications in patients undergoing lung resection. Such an association has been reported for other types of surgery, including cardiac surgery, abdominal surgery, and esophageal surgery.^7^ ^-^ ^10^

The present study allowed us to establish sex-specific 30STS thresholds for a Portuguese population, which we believe to be representative. However, we emphasize the need to apply the 30STS as a predictor of postoperative complications in populations with a higher number of women. Furthermore, future studies using a similar methodological approach should include shorter sit-to-stand assessments of 5 and 10 repetitions. If the same predictive value for postoperative complications is confirmed, its applicability could be extended. 

The results of the present study reinforce the importance of pulmonary rehabilitation, including exercise training, in the preoperative period in selected patients. The 30STS provides a noninvasive and easily reproducible method of assessing the risk of postoperative complications in the preoperative appointment, allowing early identification of patients at a higher risk of complications. Once closer monitoring and intervention protocols are established for this subgroup of patients-including tailored pulmonary rehabilitation-it will be possible to deal with potential complications earlier, thus reducing morbidity and mortality, as well as hospitalization costs. 
